# Asthma in the Precision Medicine Era: Biologics and Probiotics

**DOI:** 10.3390/ijms22094528

**Published:** 2021-04-26

**Authors:** Chiao-Juno Chiu, Miao-Tzu Huang

**Affiliations:** 1 Graduate Institute of Clinical Medicine, School of Medicine, National Taiwan University, Taipei 100, Taiwan; y4546@ms22.hinet.net; 2 Department of Medical Research, National Taiwan University Hospital, No. 7 Chung-Shan South Road, Taipei 100, Taiwan; 3 Department of Pediatrics, National Taiwan University Hospital, No. 7 Chung-Shan South Road, Taipei 100, Taiwan

**Keywords:** asthma, precision medicine, asthma endotype, biologic therapy, microbiota, probiotics

## Abstract

Asthma is a major global health issue. Over 300 million people worldwide suffer from this chronic inflammatory airway disease. Typical clinical symptoms of asthma are characterized by a recurrent wheezy cough, chest tightness, and shortness of breath. The main goals of asthma management are to alleviate asthma symptoms, reduce the risk of asthma exacerbations, and minimize long-term medicinal adverse effects. However, currently available type 2 T helper cells (Th2)-directed treatments are often ineffective due to the heterogeneity of the asthma subgroups, which manifests clinically with variable and poor treatment responses. Personalized precision therapy of asthma according to individualized clinical characteristics (phenotype) and laboratory biomarkers (endotype) is the future prospect. This mini review discusses the molecular mechanisms underlying asthma pathogenesis, including the hot sought-after topic of microbiota, add-on therapies and the potential application of probiotics in the management of asthma.

## 1. Pathophysiology of Asthma

Asthma is one of the most common chronic diseases in industrialized countries. According to the 2016 Global Burden of Disease Study, it was estimated that more than 339 million people worldwide suffer from asthma, representing a notable 3.6% increase in age-standardized prevalence since 2006 [1]. Asthma is not just a public health problem for high-income countries; it occurs in all countries regardless of the level of economic development. There were 417,918 deaths due to asthma at the global level, and most asthma-related deaths occur in low- and lower-middle-income countries [1]. Asthma symptoms most commonly develop for the first time in early childhood, but no more than half of them go on to have characteristic asthma at school age. The prevalence of asthma-like symptoms in children are varied widely between countries. The global prevalence of current wheeze in adolescents and children was estimated to be 14.1% and 11.7%, with a mean increase of 0.06% and 0.13% per year, respectively, whilst the highest prevalence of over 20% was observed in higher-income countries in both age groups [2,3]. New data on asthma prevalence and severity in children, adolescents and adults around the world are pending to be reported this year by the Global Asthma Network.

Wheezy respiration, coughing, chest tightness and shortness of breath are characteristic symptoms during asthma exacerbations. The dogma of asthma pathogenesis is that the aberrant airway epithelial sensing of environmental harmless antigens, such as pollens, mites or cockroaches or certain occupational exposures, triggers release of inflammatory mediators from the epithelia, such as thymic stromal lymphopoietin (TSLP), interleukin (IL)-25 and IL-33, which, through a cascade of mucosal immune activation involving dendritic cells (DCs), innate lymphoid cells, eosinophils, mast cells, the adaptive type 2 helper T cells (Th2) and the nerves innervating the airways, lead to tissue structural cell activation and remodeling of the endothelial cells, the goblet cells and airway smooth muscles. Recurrent allergen stimulations elicit chronic allergic inflammation of the airways, and result in irreversible structural changes and permanent lung function loss [4]. Most childhood asthma is caused by Th2-mediated airway inflammation. Th2-cell activation produces a series of cytokines. Among them, the most investigated are IL-4, IL-5, IL-9 and IL-13. IL-4 induces B-cell immunoglobulin (Ig)G to IgE class switch and IgE synthesis [5]. IL-5 enhances eosinophil proliferation and differentiation in the bone marrow, and promotes eosinophil tissue trafficking, activation as well as survival [6,7]. IL-9 supports mast-cell growth and modulates the property of type 2-driven inflammation [8,9], whilst IL-13 activates epithelial expression of inducible nitric oxide synthase (iNOs) [10], induces goblet cell hyperplasia [11] and mucus production [12], airway hyper-responsiveness (AHR) and fibrosis, bridging allergic inflammatory cells to structural non-immune cells [13]. 

The Th2-allergy paradigm came from observations predominantly made in western high-income countries. The association between allergy and asthma is not as strong in low- or middle-income countries. Occupational causes of asthma often do not involve allergy. It is now widely conceived that no more than half of asthma is caused by allergic mechanisms. In many people, asthma is caused by non-allergic inflammation of the airways, although the mechanisms involved are not yet completely understood (Table 1).

## 2. Asthma Endotypes 

Eosinophils have been long considered pathognomonic in Th2-mediated asthma; however, clinical trials have found weak correlation between eosinophilic inflammation and AHR [21,22]. For example, although anti-IgE antibody omalizumab [23] and anti-IL-5 antibody mepolizumab [24,25] reduced airway eosinophilia, they failed to show a significant effect on AHR. In contrast, anti-TNF antibody etanercept improved lung functions and reduced AHR in refractory asthma without affecting eosinophils or neutrophils [26]. In addition, patients with severe asthma may present with persistent eosinophilic inflammation in the absence of specific IgE [27,28]. Phenotypic heterogeneity of asthma and the variable treatment responses to Th2-directed trials and therapies have brought out the speculation of non-Th2 inflammation in the pathogenesis of allergic airway inflammation; the categorization of asthma based on distinct mechanistic pathways was proposed as an algorithm to tailor treatment strategies [29,30,31,32]. With the intention to leverage laboratory evidence for precision asthma treatment, two major asthma endotypes have been described (Table 1). The long-known Th2-high endotype is characterized by increased eosinophils in the sputum and airways of patients [17], whereas the Th2-low endotype manifests with increased neutrophils or a pauci-granulocytic profile [18,19,20]. 

Asthma endotype classifications combined with specific biomarkers hold great potential for new therapeutic modalities and better treatment efficacies [33]. Whilst a standardized protocol is still lacking, endotype classification is often performed according to the absolute blood/sputum eosinophil counts, serum total IgE, fractional excretion of nitric oxide (FeNO), and various allergen sensitization tests. For example, high levels of IgE, FeNO and eosinophils are biomarkers indicative of Th2-high asthma [34,35,36]. In accordance with this protocol of stratification, most of the archetypal childhood asthma falls into the Th2-high endotype, characterized by atopy, elevated IgE and FeNO levels, as well as increased sputum and blood eosinophils [37,38]. In contrast, Th2-high airway inflammation is observed in only half of adult patients with mild to moderate asthma [29], whilst among patients with moderate to severe disease, only one third of them are driven by Th2-type inflammation [39]. Th2-low endotype is characterized by neutrophil-dominated or pauci-granulocytic inflammation with high levels of IFN-γ, IL-17A/F and IL-17A/IL-22 cytokines released from Th1, Th17 or type 3 innate lymphoid cells (ILC3) [40,41]. Patients with Th2-low asthma are prone to respond poorly to corticosteroid therapy. The cause of neutrophilic inflammation in Th2-low asthma is still unclear. The nature of the inciting agents/allergens and the immediate downstream signaling pathways as elicited by the inciting agents, such as differential Notch receptor-ligand pair signaling, are tentative regulators for Th2-low inflammation [42,43]. Nonetheless, the off-target effect of high-dose corticosteroid on neutrophilia and the masking of eosinophilic inflammation by steroid therapy should be taken into consideration in Th2-low asthma presented as neutrophilic inflammation. Biomarkers for Th2-low asthma have not been widely investigated. Neutrophils, matrix metallopeptidase 9 (MMP-9) and IL-6 have all been proposed as potential candidates, but none have been shown to represent all phenotypic subgroups of the Th2-low asthma endotype [29,32]. Application of multi-omics approaches encompassing transcriptomics, epigenomics, metagenomics, metabolomics, and proteomics, in combination with clinical features and laboratory data will enable asthma endotyping to be more informative and allow the designation of precision treatment strategies [44,45].

## 3. Add-On Therapy 

A stepwise adjustment for asthma medication on an as-needed basis is suggested by the Global Initiative for Asthma (GINA) guideline [46]. The use of an inhaled corticosteroid (ICS) is an effective controller strategy in long-term management of asthma. However, the prevalence of severe or uncontrolled asthma despite good adherence to GINA-guided treatments are still as high as 10–19.8% [47,48]. For patients with poorly controlled asthma despite high-dose ICS in combination with add-on long-acting beta-agonists (LABAs), an alternative approach guided by the underlying inflammatory pathways, i.e., the endotypes, is mandatory. Add-on therapies using pharmacological non-biologic agents or biologics have been shown to improve symptom control and provide a dose-reduction strategy to limit the side effects of corticosteroid therapy [49,50,51]. Conventional add-on therapies include LABAs, long-acting muscarinic antagonists, leukotriene receptor antagonists, anti-fungal agents, macrolides and theophylline. The more recent and still rapidly progressing add-on therapies are biological agents, such as monoclonal antibodies specifically targeting inflammatory molecules. In this article, we review the add-on biologics currently available and the promising ones under clinical trials. 

## 4. FDA-Approved Monoclonal Antibodies 

There are currently five biologics licensed for severe asthma in adults: omalizumab binds to IgE at Fc𝛆RI binding site; mepolizumab and reslizumab both bind to IL-5; and benralizumab binds to IL-5 receptor α subunit and dupilumab, which binds to IL4 receptor α subunit, thus blocking both IL-4 and IL-13 signaling (Table 2).

Omalizumab is the first biologic approved for the treatment of asthma in the U.S. and European Union. Omalizumab is a humanized recombinant monoclonal antibody with binding specificity at the FcεRI binding site of IgE, thus preventing IgE binding to Fc𝛆RI on mast cells, basophils and DCs, and the subsequent release of inflammatory mediators from these cells. It reduces asthma exacerbations and the maintenance doses of ICS and improves quality-of life scores in clinical trials [18,69,70]. Omalizumab is currently specifically indicated for moderate-to-severe persistent asthma with serum IgE greater than 30 IU/L in both adults and children 6 years of age and older. In adolescent and adult severe asthmatic patients, omalizumab has shown beneficial real-world short-term effectiveness at 1 year and strong evidence of long-term effectiveness for up to 4 years and beyond [71]. 

There are three licensed biologics targeting IL-5-mediated inflammatory pathway, including mepolizumab, reslizumab, and benralizumab. Mepolizumab and reslizumab are humanized monoclonal antibodies specifically targeting IL-5. Whilst reslizumab is administered by intravenous infusion with weight-based dosing [56], mepolizumab is delivered in a fixed dose of 100 mg subcutaneously to patients aged 12 years and above. In young children 6–11 years of age, mepolizumab is used at a lower dose of 40 mg [72,73,74]. In oral corticosteroid-dependent patients, mepolizumab reduces the need for oral corticosteroid therapy [75]. Reslizumab reduces the exacerbation frequency and improves lung function [76,77]. The other biologic targeting IL-5 signaling pathway is benralizumab. It binds to the IL-5 receptor on eosinophils and basophils, and thus prevents binding of the IL-5 receptor by IL-5 [78]. A systematic review and network meta-analysis showed that reslizumab may be more effective than benralizumab in patients with eosinophilic asthma receiving GINA step4/5 treatment [79].

Dupilumab was approved by the FDA for the treatment of adult moderate-to-severe atopic dermatitis in 2017, and later for moderate-to-severe asthma in 2018 [80]. It inhibits both IL-4 and IL-13 signaling and reduces Th2 response through direct binding to the IL-4R⍺, the shared subunit for IL-4 and IL-13 receptors, hence preventing IL-4 and IL-13 interaction with the receptors. Dupilumab notably reduced serum total IgE levels and FeNO and increased blood eosinophil counts. The increase in blood eosinophils is plausibly attributed to the blockade of IL-4 and IL-13 effects on eosinophil survival, activation, and tissues trafficking by dupilumab, but not mobilization of eosinophils from bone marrow, which is influenced by IL-5. Dupilumab reduced the exacerbation risk of severe asthma, and improved FEV1 without an increased risk of adverse effects [81]. Comorbidities, including atopic dermatitis, chronic rhinosinusitis, and allergic rhinitis, may also respond to treatment with dupilumab [82].

The data on biologic therapies are mostly derived from studies on adults; it is extremely limited in the pediatric population, and even more limited in children younger than 12 years of age. As of March 2021, the FDA has approved four biologic drugs for use in pediatric patients with severe asthma: omalizumab, mepolizumab, benralizumab and dupilumab. Whilst omalizumab and mepolizumab are the only two biologics approved for children 6–18 years of age, benralizumab and dupilumab are licensed for use in adolescents aged 12 years and older [83,84]. 

## 5. Biological Therapies under Clinical Trials 

Currently available biologic therapies, including anti-IgE, anti-IL-5, anti-IL-5R⍺ and anti-IL-4R⍺, reduce asthma exacerbation rates in patients with Th2-high asthma. However, there are no effective treatments for patients with severe Th2-low asthma. The airway epithelium acts as the first line of defense against airborne substances. The classic features of asthma exacerbations are initiated by the releasing of alarmins, including TSLP, IL-33 and IL-25, from the airway epithelium in response to inflammation or injury [85]. These cytokines activate group 2 innate lymphoid cells (ILC2), which produce large amounts of IL-5, IL-13, and a small amount of IL-4 without production of specific IgE [13]. These findings may partially explain why patients with severe asthma lack an allergen-induced Th2 response, but manifest with persistent eosinophilic inflammation. Currently, clinical trials for biologics antagonizing alarmins include tezepelumab against TSLP, etokimab for inhibiting IL-33, and lebrikizumab for IL-13 blockage (Table 1). 

Additionally, for alleviating airflow obstruction by preventing airway smooth muscle contraction, a new biologic GDC-0334 is under trial for its effects in inhibiting transient receptor potential cation channel member A1 (TRPA1) activation. TRPA1 is a nonselective cation channel, monitoring changes in the chemical environment, and responds to physical stimuli, such as mechanical stress or changes in temperature [86]. The conditional deletion of TRPA1 in neuronal cells resulted in reduced inflammatory cell infiltration and IL-5 production [87]. These findings indicate that neuronal TRPA1 is critical in asthmatic inflammation. Recent preclinical studies showed that TRPA1 blockage with a small molecule inhibitor GDC-0334 suppressed inflammation and airway smooth muscle contraction [88]. Instead of direct blockage of the immunologic factors mediating asthma pathogenesis, alternative approaches targeting pathogenic factors, such as those involved in neurogenic inflammation in asthma, hold great potential for the treatment of Th2-low asthma.

## 6. Microbiota and Allergic Asthma 

### 6.1. Hygiene Hypothesis 

The seminal study linking microbial exposures with the tendency of developing allergic diseases was conducted by the British epidemiologist Professor David Strachan over 30 years ago. The theory he proposed is nowadays widely known as the “hygiene hypothesis” [89]. According to the theory, reduced exposures to environmental bacteria in early life, including birth by cesarean section, being bottle-fed, growing up in the city, fewer family members or contacts to various persons and less infections due to vaccinations, are associated with an increased risk of developing allergies and asthma later in life. The mechanistic thinking derived from the hygiene hypothesis is that microbial exposures during the perinatal stage influence the establishment of a child’s gut microbiota. Microbial alterations, i.e., dysbiosis, driven by these “hygienic” factors, acting through affecting the infant immune development and responses, are causally related to the increased risks of allergic diseases [90,91]. 

In line with this hypothesis, studies have shown that children growing up in developed countries or in urban areas, where allergies are more prevalent, host different gut microbiota compared to children growing up in underdeveloped countries or in farm fields, where allergies are relatively rare [92,93,94]. In addition, phylogenetic differences in the home microbiota in early life were associated with a subsequent risk of childhood asthma [95]; farm-like indoor microbiota has been shown to protect children living in non-farm homes from developing asthma, suggesting that the indoor dust microbiota composition could not only be a predictor of asthma risk, but also pose as a potential modifiable target for asthma prevention [93]. 

### 6.2. Gut–Lung Axis and Microbial Mechanisms

With intensive research in the field of microbiota over the past decade, including the completion of the NIH-funded Human Microbiota Project (HMP) [96,97], we have come to appreciate more the role of microbiota in maintaining health and that alterations in the gut microbiota, i.e., dysbiosis, not only causes perturbations of the immune responses within the guts, but it also impacts on the well-being of distant organs, such as the lungs [98]. The concept for the intricate and reciprocal interactions between the gut and lungs, i.e., the gut–lung axis, was prompted by the observation that changes in the intestinal milieu influenced or primed the progress of different lung diseases and vice versa. Whilst how communications between the gut and lungs are achieved are still not completely understood, it has been suggested and well-accepted that mediators derived from intestinal epithelial cells, immune cells, the microbial structural components and/or microbial metabolites traffic through circulation and elicit changes in immune response in the lungs. One good example demonstrating the role of microbiota in the gut–lung axis is the studies on abnormal secretory IgA (SIgA) microbial binding. SIgA is the first line of defense of the mucosa against tissue invasion by pathogens and commensal bacteria. SIgA limits the overgrowth of microbial species and hence guards the compositions and properties of the microbiota. In this regard, studies have found that children with a lower IgA binding to fecal bacteria at 12 months of age are more likely to develop asthma and allergic diseases [99]. Interestingly, altered IgA recognition patterns in children with allergies were observed at ages as early as 1 month old, when IgA in breast-fed children are predominantly maternally derived. Whether it indicates a dysbiotic state of the mothers warrants more investigation. 

Mammals harbor over 100 trillion gut bacteria from over 1000 different species. Commensal gut floras have been shown to induce the differentiation of particular CD4^+^ T cell subsets. Examples include the induction of Th17 cells in the intestinal lamina propria by segmented filamentous bacteria (*SFB*) [100], the development of systemic Th1 cells [101] and local IL-10-producing Tregs [102] by *Bacteroides fragilis* and the induction of colonic Treg cells by indigenous *Clostridium* species [103]. Whilst cumulating data point to a crucial role of the commensal microbes in shaping and regulating the immune system [104,105,106,107,108], the mechanisms underpinning this function are only gradually being uncovered.

In the context of the gut–lung axis in pathogenesis of allergic airway diseases, a series of studies have elegantly demonstrated that short-chain fatty acids (SCFAs), acetate, propionate, and butyrate, the metabolic products of microbial fermentation of indigestible dietary fibers, promoted not only colonic but also peripheral Treg expansion [109,110,111,112]. 

Metabolites derived from gut microbial functions circulate systematically to distant organs, including the lungs, and regulate the pathophysiological status therein, and vice versa. In a murine asthma model, high-fiber diets increased circulating levels of SCFAs and protected the mice against allergic inflammation in the lungs [113]. Treating the mice with the SCFA propionate recapitulated the protective effect of a high-fiber diet against allergic airway inflammation. In vivo, propionate enhanced the generation of macrophage and DC precursors and subsequent trafficking of these cells to the lungs. However, propionate-induced DCs were ineffective in promoting Th2 effector function [113]. In agreement with these findings, others have shown that butyrate inhibited pulmonary ILC2 functions and the subsequent development of airway hyperreactivity (AHR) through modulation of GATA3 expression and metabolic pathways of pulmonary ILC2s. Association of germfree mice with butyrate-producing gut bacteria effectively suppressed ILC2-driven AHR [114]. These studies highlighted the beneficial effects of high-fiber diets and SCFAs in the prevention of asthma. 

SCFAs are pleiotropic metabolites implicated in an array of physiological processes, including the production of satiety hormones, GLP-1, PYY, and leptin, energy expenditure, epithelial proliferation and epithelial barrier function [115]. In addition, SCFAs inhibit LPS-induced NF-kB activation in neutrophils and macrophages by binding to receptors GPR41, GPR43, and GPR109A and by inhibition of histone deacetylase [109,110,111]. With the various beneficial effects of the SCFAs, development of microbiota-directed food or fiber-based interventions to promote growth of SCFA-producing microbiotas provides an alternative preventive and/or therapeutic modality for asthma.

### 6.3. Relation of Microbial Taxa and Asthma 

The burgeoning of microbial studies in recent years is in a large part attributed to the revolutionary advances in metagenomic sequencing, bioinformatics and multi-omics technologies, which allow detailed analysis and identification of the non-culturable microorganisms, in addition to the culturable ones, and their biological products [92]. Previously unrecognized and underappreciated functions of the microbiota in shaping the immune systems and in the pathogenesis of diseases, not only of the gastrointestinal tract, but also of the distant organs, including the lungs and the brain, have been uncovered. Recent studies have elegantly shown a critical window of life, during which the microbiota contributes to education and maturation of the immune system, facilitating the establishment of tolerance to environmental harmless exposures or perpetuate the development of disease later in life [116]. 

Early-life airway microbiota may predispose to the development of asthma in childhood through dynamic interactions with the developing immune system [117]. Altered compositions of the airway [118,119,120] and gut microbiota [121,122,123] have both been linked to higher risk of atopy and asthma [124,125,126]. Collectively, increased relative abundance of *Bacteroidaceae*, *Clostridiaceae*, and *Enterobacteriaceae* and a lower abundance of *Bifidobacteriaceae* and *Lactobacillaceae* are associated with the development of allergic sensitization, eczema, or asthma [127], whereas members of the *Lachnospiraceae* family and the genera *Faecalibacterium* and *Dialister* are protective of developing atopy [128]. In one study, a group of neonates with highest risk of developing atopy and asthma were identified when the stool microbiome contained lower relative abundance of *Bifidobacterium*, *Akkermansia* and *Faecalibacterium* and higher relative abundance of *Candida* and *Rhodotorula* [129]. Fecal metabolome from these high-risk neonates showed enriched pro-inflammatory metabolites, such as 12, 13-DiHOME. Both 12, 13-DiHOME and fecal water from these neonates were able to induce IL-4-producing T cells and concomitantly reduced FoxP3^+^ regulatory T cell differentiation. These findings plausibly support that dysbiosis perturbs the immune system resulting in pathogenic T cell dysfunction that causes atopy and allergic diseases [129].

In addition to predilection to atopy and allergic diseases, dysbiosis may also hamper therapeutic efficacies [130,131]. An analysis of the bronchoalveolar lavage microbiome found distinct microbial expansions in patients with corticosteroid resistant (CR) asthma [130]. Among them, *Haemophilus parainfluenzae* was uniquely expanded only in CR asthma airways. Incubation of asthmatic airway macrophages with *H. parainfluenzae* resulted in TAK1/MAPK activation and corticosteroid resistance. 

### 6.4. Contradictory Data—Take Ruminococcus gnavus as an Example 

In a Canadian cohort of infants, bacterial genera *Lachnospira*, *Veillonella*, *Faecalibacterium*, and *Rothia* were found to be significantly decreased at 3 months of age in children who later developed atopic asthma [122]. The causal effects of these bacterial taxa in preventing asthma development were further verified in an animal model of asthma, wherein offspring of the gnotobiotic mice harboring these four bacterial taxa were able to resist allergen-induced airway inflammation. In a rural Ecuadorian cohort investigated by the same group, increased relative abundance of *Streptococcus* and *Bacteroides* species and decreased *Bifidobacterium* species and *Ruminococcus gnavus* at 3 months of age were associated with a higher risk of atopy and asthma at 5 years old [121]. The gut microbiota regulates immune responses not only locally but also in distal organs at least partly through microbial metabolites. In a recent study, microbial bile acid metabolism has been linked with Foxp3^+^ Treg-cell induction [132]. Ruminococcus *gnavus* is in a particularly important position in bile acid-elicited immune regulation for its capacity to epimerize 3α-hydroxydeoxycholic acid (DCA) to 3β-hydroxydeoxycholic acid (isoDCA), the most potent de-conjugated bile acid to induce colonic Treg differentiation [133]. These findings plausibly provide the mechanistic ground for the association between decreased *R. gnavus* abundance and increased atopy and asthma risk in previous studies [121,134]. In contradiction to the studies presented above, a twin cohort study has found an association between increased relative abundance of *Lachnospiraceae* at 2 months of age and a higher risk of developing allergic diseases before the age of 3 [123]. In this study, *R. gnavus* was the dominant responsible species for increased allergy risk. When conventional naïve mice and allergen-sensitized mice were colonized with *R. gnavus*, an enhanced allergic airway inflammation was observed.

Whilst experimental settings, geographic, genetic and cultural differences among individual study cohorts may underlie the non-concordant data seen in different studies, interpretation of these data cannot, nonetheless, be too cautious. The quality or performance of the metagenomics platforms as well as the physiological fidelity of the animal models in microbial reconstitution should all be carefully evaluated. In this aspect, colonization of the microbes of interest in conventional specific pathogen-free (SPF) mice may carry the risk of perturbing the gut commensal community and inducing advert immune responses to the microbes by itself. In addition, the timing for colonizing the mice with the microbes, e.g., before allergen sensitization occurs or after, should also be taken into account to justify the rationales underlying the hypothesis with regard to the role and function of the microbiota in the pathogenesis of allergic diseases. To date, the optimal physiological way to avoid these untoward effects is by using the offspring of the gnotobiotic mice inoculated with the microbe of interest; the offspring are borne to parents carrying these microbes and are, therefore, colonized by these microbes in a more physiological way. 

### 6.5. Therapeutic Potential of Microbiota

#### 6.5.1. Probiotics

Randomized controlled trials (RCTs) for using probiotics or in combination with prebiotics in asthma prevention and control have shown mixed efficacy outcomes. For this reason, several meta-analysis studies have been performed. We searched PubMed for meta-analysis studies with the keywords, probiotics, microbiota, asthma, allergies and atopy from 2010 to 2021. Four meta-analysis studies analyzing published trials from inception to 2013–2018 with detailed descriptions for inclusion/exclusion criteria, methods of analysis and analysis results were selected for review [135,136,137,138]. Collectively, these meta-analyses have pointed to a concordant conclusion that there is no evidence to support prenatal or postnatal administration of probiotics as a standard asthma prevention strategy based on the RCT data published thus far. Although in some analyses, prenatal and/or early-life probiotic supplementations did show protective association with decreased atopic sensitization, IgE production and infantile eczema, they did not necessarily exert beneficial effects in asthma prevention or wheeze risk [136,137,139,140]. 

One recent study published in 2020 analyzed 30 RCTs dated from 2003 to 2018, investigating the effects of probiotic supplements for asthma risk (primary outcome) or wheeze incidence (secondary outcome) in infants [138]. The probiotics applied in these trials included Lactobacillus (L.) *reuteri*, *L. rhamnosus GG*, *L. rhamnosus LC705*, *L. acidophilus*, *L. paracaseii*, *L. casei*, Bifidobacillus (B.) *lactis*, *B. bifidum*, *B. breve Bbi99* plus Propionibacterium *freudenreichii ssp shermanii*, and B. *longum BL999*. The probiotics were administered either alone or in combination with prebiotics as postnatal interventions or started since the prenatal stage. No significant association of probiotic supplementations with lower asthma risk or wheeze incidence was found [135,140,141,142,143,144,145,146,147,148,149,150,151,152]. In contrast, in subgroup analyses by asthma risk, probiotic supplementations significantly reduced wheeze incidence among infants with atopy, whilst there were still no significant associations in infants with other asthma risk factors, such as family history or a cow’s milk allergy [140,144,145,146,147,153,154,155,156,157,158,159,160,161]. 

Therefore, despite various studies that have demonstrated a crucial and beneficial role of microbiota in modulating the immune responses and preventing atopy and allergic diseases, the use of probiotics as a therapeutic strategy for asthma is not, as of yet, conclusive. Nonetheless, dietary fiber is nowadays regarded as part of a healthy diet worldwide, and development of dietary fiber-based interventions, which selectively increase the abundance of microbes, that provide metabolic benefits to the host, such as SCFA production, is actively underway [162,163,164].

#### 6.5.2. From Microbial Endotypes to Asthma Endotyping and Precision Medicine for Asthma

The heterogeneity of asthma does not confine to diverse clinical phenotypes and aetiologies; it also manifests with different airway microbiomes. Both bacterial and fungal microbiota signatures were found to correlate with asthma endotypes and clinical features. In recent studies employing the omics approach, decreased airway bacterial and fungal diversity as well as increased relative abundance of *Pseudomonas*, *Trichoderma*, *Fusarium*, *Cladosporium* and *Aspergillus* were associated with Th2-high endotype, whereas increased *Proteobacteria*, *Mycosphaerella* and *Penicillium* were clustered with Th2-low type of asthma [119,165,166]. The association of microbiome endotypes with asthma endotypes may further contribute to precision asthma endotyping and selection of treatment regimens; however, it has to be taken into consideration that concurrent steroid treatment may change the microbiome and obscure the true association [167].

The microbiome has been shown to affect corticosteroid responsiveness in asthma [130,131]. It is largely unknown whether the microbiome also affects treatment efficacies of the biologics targeting specific features of asthma-related immune mechanisms. In one such study using nasal secretion samples collected from asthmatic children enrolled in an omalizumab trial, nasal *Moraxella* species was found to associate with increased asthma exacerbations and eosinophil activation. [168]. Therefore, whilst omalizumab successfully reduced asthma exacerbations, the nasal airway microbiota composition might remain largely unaffected. The persistence of pathogenic nasal airway microbiota, such as the *Moraxella* species, which occurred more often in young children and caused increased epithelial damage and eosinophil activation, is capable of evading targeting biologic therapies. 

The findings from these studies indicate that the importance of microbiota goes beyond influencing the development of atopy and asthma at the early life, and the application of microbiota is not restricted to probiotics. Instead, incorporation of microbiome endotypes of individual asthmatic patients can further stratify asthma endotyping and enable the identification of pathogenic microbiome endotypes, which collectively warrant optimal treatment regimen tailoring. Future precision asthma therapies based on microbiome-associated asthma endotypes may potentially comprise treatment targeting pathogenic or dysbiotic microbiota and the biological therapies targeting the underlying inflammatory processes, in addition to pharmacological drugs.

## 7. Conclusions

**Figure 1:**
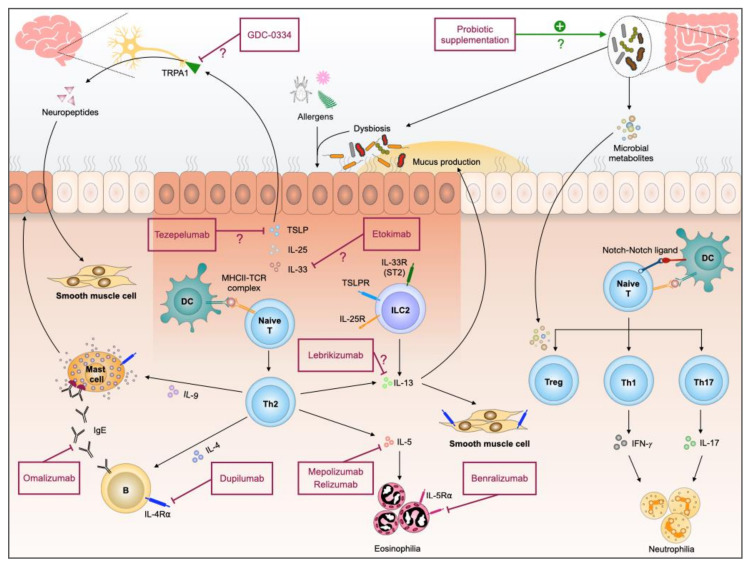
Pathogenesis of allergic airway inflammation and therapeutic interventions currently available and those with potential.

With advances in metagenomics, bioinformatics and single-cell platforms, we are witnessing a rapid progress in translational research. In the context of allergic airway diseases, it has become even more clear the heterogeneity of asthma, attributing in a large part to the complex interactions between the host and the environment, including the microbial community: those existing in the environment in which the host lives and those inhabiting the host. Re-classification of asthma into distinct endotypes in a laboratory- and clinical-evidence-based manner contributes to personalized precision medicine. In addition, distinct microbiome endotypes and asthma endotypes’ association suggests the potential utilization of microbiome-targeting as a novel add-on therapy strategy in precision asthma treatment. Whist we understand more of the Th2-high asthma and successfully introduce novel biologics as a Th2-high therapeutic strategy, more investigation is needed for Th2-low asthma before we can leverage the advances in scientific research in designing optimal therapies for these patients (Figure 1).

## Figures and Tables

**Table 1:** Clinical and laboratory characteristics differentiating allergic vs. non-allergic asthma.

	Allergic Asthma (Th2-High)	Non-Allergic Asthma (Th2-Low or Non-Th2)
Prevalence [14]	60%	10–33%
Age of onset	Occurs early in life	Mostly occurs later in life
Triggers	House dust mites, pollen, pet dander and cockroaches, etc.	More diverse. Cold air, smoke, obesity, occupational exposure, and exercise
Inflammatory mediators	IL-4, IL-5, IL-13, IL-25, IL-33 and TSLP	IL-1β, IL-6, IFN-γ, TNF-α and IL-17
Severity	Milder than Th2-low	More severe than allergic asthma
Treatment	Responds well to ICS	Require higher doses of ICS or non-responsive to ICS [15,16]
Recruiting cells in the airway	Eosinophilic inflammation [17]	neutrophilic or pauci-granulocytic inflammation [18,19,20]
Serum total IgE	High	Normal
Skin prick test	Positive	Negative

ICS: inhaled corticosteroids.

**Table 2:** Monoclonal antibodies and small molecule drugs for allergic asthma treatment.

FDA-Approved Monoclonal Antibodies								
*Name*		*Target*	*Biological Effects*		*Dosing*		*Indication*	*Sponsor*
Omalizumab (Xolair^®^)		FcεRI binding site of IgE [52,53]	Decrease circulating total IgE Decreased expression of FcεRI on inflammatory cells Decreased mediator release.		According to IgE levels Every 2–4 wks (s.c.)		Moderate-severe allergic asthma IgE ≥ 30 IU/mL + skin prick test	Genentech
Mepolizumab (Nucala^®^)		IL-5 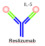	inhibiting the bioactivity of IL-5 by blocking its binding to IL-5Rα complex expressed on the eosinophil cell surface [54] Reduces the production and survival of eosinophils		100 mg Every 4 wks (s.c.)		Severe eosinophilic asthma blood eosinophils ≥ 400/uL	GlaxoSmithKline
Reslizumab (Cinqair^®^)		IL-5 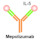	Inhibiting IL-5 signaling [55] Decreased eosinophils in blood and sputum [56]		3 mg/kg Every 4 wks (i.v.)		Severe eosinophilic asthma blood eosinophils ≥ 400/uL	Teva Pharmaceuticals
Benralizumab (Fasenta^®^)		IL-5R⍺ 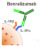	Decreased eosinophils and basophils though ADCC [57]		30 mg Every 8 wks (s.c.) [58,59]		Severe eosinophilic asthma blood eosinophils ≥ 300/uL	AstraZeneca
Dupilumab (Dupixent^®^)		IL-4R⍺	Blockade IL-4/IL-4R⍺ binding Blockade IL-13/IL-4R⍺ binding		300 mg Every 2 wks (s.c.) [60]		blood eosinophils ≥ 150/uL FeNO > 25 ppb	Sanofi and Regeneron
**Biological Therapies under Clinical Trials**								
***Name***	***Target***		***Biological Effects***	***Dosing***		***Indication***	***Sponsor/Development Status***	
Tezepelumab	TSLP		TSLP blockade [61,62,63]	210 mg/kg Every 4 wks (s.c.) [64]		Patients with high (≥300 cells/µL) or low (<300 cells/µL) blood eosinophil counts Adults with oral corticosteroid-dependent asthma	AstraZeneca and Amgen/Phase III (NCT03406078) https://clinicaltrials.gov/ct2/show/NCT03406078 (accessed on 20 April 2021)	
Etokimab (ANB020)	IL-33		IL-33 blockade	300 mg single dose (i.v.)		Adults with severe eosinophilic asthma blood eosinophil counts ≥ 300 cells/µL Stably maintained on ICS/LABA dose for at least 3 months [65]	AnaptysBio/Phase IIa(NCT03469934) https://clinicaltrials.gov/ct2/show/NCT03469934 (accessed on 20 April 2021)	
Lebrikizumab (RO5490255)	IL-13		Binds to soluble IL-13 and blocks downstream signaling [66,67]	125 mg Every 4 wks (s.c.) [68]		Adults with uncontrolled asthma On ICS and a second controller medication	Hoffmann-La Roche/Phase II (NCT02099656) https://clinicaltrials.gov/ct2/show/NCT02099656 (accessed on 20 April 2021)	
GDC-0334 (small molecule)	TRPA1		TRPA1 inhibitor	Orally with dose escalation between cohorts		Phase I study in healthy adult subjects	Genentech/Phase I (NCT03381144) https://clinicaltrials.gov/ct2/show/NCT03381144 (accessed on 20 April 2021)	

The table includes published data of approved therapies and clinical trials from the database of ClinicalTrials.gov (provided by the U.S. National Library of Medicine). The clinical trials were searched with MeSH keywords, including condition or disease—Asthma; study type—interventional studies; status—recruiting, enrolling by invitation, active, suspended, and completed from inception to March 2021.

## Data Availability

Not applicable.
